# Molecular Diagnosis of Analbuminemia: A New Case Caused by a Nonsense Mutation in the Albumin Gene

**DOI:** 10.3390/ijms12117314

**Published:** 2011-10-25

**Authors:** Monica Dagnino, Gianluca Caridi, Ueli Haenni, Adrian Duss, Fabienne Aregger, Monica Campagnoli, Monica Galliano, Lorenzo Minchiotti

**Affiliations:** 1 Laboratory on Pathophysiology of Uremia, Istituto Giannina Gaslini IRCCS, Genova 16148, Italy; E-Mails: monicadagnino@ospedale-gaslini.ge.it (M.D.); gianlucacaridi@ospedale-gaslini.ge.it (G.C.); 2 Praxis Kreuzmatte, Kreuzstrasse 2, Postfach, 3052 Zollikofen, Switzerland; 3 Department of Nephrology/Hypertension, Inselspital, Bern University Hospital, and University of Bern, Bern 3010, Switzerland; E-Mails: adrian.duss@ksl.ch (A.D.); fabienne.aregger@charite.de (F.A.); 4 Department of Biochemistry “A.Castellani”, University of Pavia, Pavia 27100, Italy; E-Mails: monica.campagnoli@unipv.it (M.C.); galliano@unipv.it (M.G.)

**Keywords:** human serum albumin, analbuminemia, heteroduplex analysis, single-strand conformation polymorphism, DNA sequence

## Abstract

Analbuminemia is a rare autosomal recessive disorder manifested by the absence, or severe reduction, of circulating serum albumin (ALB). We report here a new case diagnosed in a 45 years old man of Southwestern Asian origin, living in Switzerland, on the basis of his low ALB concentration (0.9 g/L) in the absence of renal or gastrointestinal protein loss, or liver dysfunction. The clinical diagnosis was confirmed by a mutational analysis of the albumin (*ALB*) gene, carried out by single-strand conformational polymorphism (SSCP), heteroduplex analysis (HA), and DNA sequencing. This screening of the *ALB* gene revealed that the proband is homozygous for two mutations: the insertion of a T in a stretch of eight Ts spanning positions c.1289 + 23–c.1289 + 30 of intron 10 and a c.802 G > T transversion in exon 7. Whereas the presence of an additional T in the poly-T tract has no direct deleterious effect, the latter nonsense mutation changes the codon GAA for Glu244 to the stop codon TAA, resulting in a premature termination of the polypeptide chain. The putative protein product would have a length of only 243 amino acid residues instead of the normal 585 found in the mature serum albumin, but no evidence for the presence in serum of such a truncated polypeptide chain could be obtained by two dimensional electrophoresis and western blotting analysis.

## 1. Introduction

Congenital analbuminemia (MIM 103600) is a very rare autosomal recessive disorder [1]. In spite of the fact that the trait is readily detected by routine plasma electrophoretic analysis, only 51 cases have been so far reported world-wide in 43 families and are listed in the continuously updated Register of Analbuminemia Cases [2]. Thus, the incidence is less than one in one million for most populations, apparently without gender or ethnic predilection. However, fetal or neonatal death of siblings was frequently observed in the families of analbuminemic subjects [2,3]. This seems to confirm the hypothesis that only a few analbuminemic individuals survive past the neonatal state and that serum albumin (ALB) may play a crucial role in fetal development [4]. In contrast, during childhood and adult life, the analbuminemic individuals usually have surprisingly minimal findings, since the absence of ALB is partially compensated for by an increase of other serum proteins, such as globulins, transferrin, and coagulation factors [1]. The main clinical symptoms include mild edema and occasionally chronic fatigue or lipodystrophy, especially in women, while the most common biochemical signs are gross hyperlipidemia with hypercholesterolemia and elevated LDL-cholesterol levels [1]. The finding of a low ALB concentration during routine serum electrophoresis, with normal liver function and no gastrointestinal or renal protein loss, suggests the clinical diagnosis. This needs to be confirmed by the molecular diagnosis, based on the identification of the causative mutation within the albumin (*ALB*) gene by DNA sequence analysis. In our continuing study of this defect, we report here a new case of analbuminemia observed in a 45-year-old man of Lebanese origin living in Switzerland and the identification of the causative mutation within the *ALB* gene.

## 2. Results and Discussion

### 2.1. Patient

The patient is a 45-year-old man, born in Beirut, Lebanon, to parents of Syrian origin, living in Switzerland. The family comes from As Suwayda, a mainly Druze town located in Southwestern Syria, close to the border with Jordan. The parents are first degree cousins, and the patient is the second among ten children (seven brothers and three sisters), who all used to live in Lebanon, but have now spread to different countries. The patient presented with severe lipodystrophy. He reported that since the age of 17 years he was suffering from edematous legs, for which he is currently under treatment by lymph drainage massage once in two weeks, plus tightly wrapped bandages. Among his brothers and sisters, only the eldest sister has also swollen legs, but, unlike our patient, she can only walk with significant limitations. As far as he can remember, his mother had no still-births or abortions or problems getting pregnant. He reported an albumin level, measured by dye binding techniques, of 16 g/L (normal reference range 37–52 g/L). His most relevant clinical chemistry data, determined at the Bern University Hospital, are summarized in Table 1.

The patient shows severe hypoalbuminemia and moderate hypoproteinemia, because of the compensatory increase of the globulin fractions. Also a western blotting analysis, performed as described in the Experimental Section, confirmed the near complete absence of ALB from the proband’s serum (data not shown). The serum lipid profile exhibits moderate dyslipidemia: total cholesterol and LDL cholesterol are elevated, whereas HDL-cholesterol and triglycerides are in the normal range. On the basis of the results of the plasma protein electrophoresis and of the western blotting analysis congenital hypoalbuminemia was diagnosed in our patient, since other more common causes of low ALB levels, such as renal or gastrointestinal protein loss, or liver dysfunction, were ruled out.

### 2.2. Mutational Analysis

**Figure 1:**
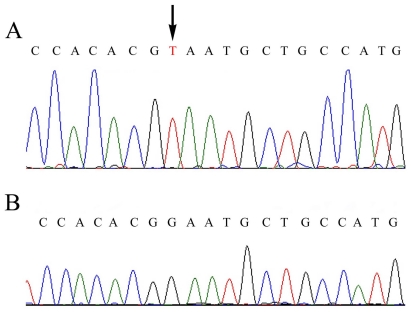
Genomic DNA sequence electropherograms showing the mutation found in exon 7 of the patient: (**A**) proband; (**B**) control for the wild type sequence. The arrows indicate the c.802 G > T transversion in exon 7. The patient is homozygous for the mutation.

To confirm this diagnosis at the molecular level, a mutational analysis of the *ALB* gene was carried out as described in the Experimental Section. Heteroduplex analysis (HA) revealed the presence of a mutation in the 406 bp long region amplified by using PCR primers A19B and A20C encompassing exon 10 and the intron9-exon10 and exon10-intron10 junctions [4]. DNA sequence of this fragment showed that the patient is homozygous for the insertion of a T in a stretch of eight Ts spanning positions c.1289 + 23–c.1289 + 30 of intron 10 [5]. This mutation, reported as rs71217465 in the Single Nucleotide Polymorphism databank at NCBI, was previously identified in another analbuminemic subject, a girl from El Jadida (Morocco), but no direct deleterious effect could be ascribed to the presence of an additional T in this poly-T tract [6]. DNA sequence analysis of the remaining thirteen coding exons and their adjacent intron regions revealed the presence of an additional mutation in the region encompassing exon 7 and the intron6-exon7 and exon7-intron7 junctions that escaped our electrophoretic analysis. The electropherogram of the proband (Figure 1B) revealed that he is homozygous for a c.802 G > T transversion in exon 7.

**Figure 2:**
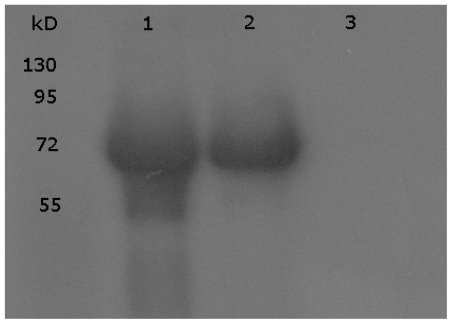
Western blotting of the patient’s serum showing the complete absence of the protein.ine 1, ALB; line 2, control serum; line 3, proband’s serum.

This nonsense mutation, that was also present in the analbuminemic girl from El Jadida [6], and, as such, is reported as pathogenic variation in the Single Nucleotide Polymorphism databank at NCBI (rs78340021), changes the codon GAA for Glu244 to the stop codon TAA, resulting in a premature termination of the polypeptide chain. The putative protein product would have a length of only 243 amino acid residues (p.Glu268X) instead of the normal 585 found in the mature serum albumin, but no evidence for the presence in serum of such a truncated polypeptide chain could be obtained by two dimensional electrophoresis (data not shown) and western blotting analysis (Figure 2).

Analbuminemia is thought to be associated with very few medical complications. However, an open question is whether analbuminemic individuals are at risk for atherosclerotic complications, considering most of them are known to have high cholesterol levels. Hypercholesterolemia may have been responsible for some cases of premature coronary heart disease, although there are very few reports of patients with congenital analbuminemia who have had long-term follow-up as adults. Therefore, although most patients with hereditary analbuminemia have not experienced premature atherosclerosis, treatment with statins appears to be recommended [7,8]. In view of the altered pharmacokinetics in patients with analbuminemia, albumin-bound drugs should be administered with caution and monitored carefully [8]. Our patient was treated for four weeks with atorvastatin (20 mg/day), but this statin was then stopped because he developed myopathy, one of the most common side effects of statins. He is currently being treated with another statin with lower plasma protein binding, such as pravastatin (40 mg/day). The proband also shows moderate hypocalcemia and hyperphosphatemia. ALB is the principal transport and depot protein for calcium in blood plasma and about 45% of circulating calcium is bound to ALB [1]. Consequently, patients with a low ALB concentration are expected to have lower serum total calcium concentrations with biological active ionized calcium in a normal range [9].

To date seventeen different mutations causing analbuminemia have been identified in the coding regions of the *ALB* gene and their intron–exon junctions in sixteen different subjects [2,3,10–12]: six nonsense mutations, four mutations affecting splicing, four frameshift/deletion, one frameshift/insertion, and a compound heterozygous nonsense/splice site mutation. The results show that analbuminemia is an allelic heterogeneous disorder caused by homozygous or compound heterozygous inheritance of defects. The molecular defects are located in eight different exons (3, 4, 5, 7, 8, 10, 11, and 12) and in three different introns (1, 6, and 11) [2,3,10–12]. Although those findings seem to suggest that analbuminemia is the result of widely scattered random mutations [10], the presence of regions in the *ALB* gene that are prone to mutations resulting in analbuminemia is still an open question. The presence of such regions has been hypothesized in the exon 11-intron 11 junction and within exon 12 [3,12].

The majority of those mutations are unique, *i.e.*, they have been found in only a single individual or in the members of the same family. Exceptions to this rule are a c.412 C > T transition, found in two unrelated individuals (analbuminemia Bethesda), and a c.228_229delAT (analbuminemia Kayseri), found in twelve subjects of ten apparently unrelated families, that therefore appears to be the most frequent cause of analbuminemia so far identified [2,3 and other cases studied in our lab]. Here, we report the second instance of a c.802 G > T transversion that was previously described in a five-year-old girl, the first child of a couple from El Jadida, Morocco [6].

As a consequence of the seventeen identified mutations, the protein product was predicted to range in length from 19 to 532 amino acid residues, although no evidence was found for the presence in serum of a truncated molecule [2,3]. A possible molecular explanation is that these putative proteins will partly or completely lack domain III of ALB, that has been shown to be crucial for binding to the neonatal Fc receptor (FcRn), which acts to recycle ALB to the serum, and therefore these truncated molecules cannot avoid lysosomal degradation [13]. Recent studies with a recombinant truncated ALB variant similar to a clinically observed splice mutant denoted Bartin, that lacks the whole of domain III except the first 25 amino acids, confirmed no detectable pH dependent FcRn binding [14].

The analbuminemic individuals are prone to misdiagnosis or delayed diagnosis. In our patient the absence of severe symptoms and the presence of a significant, though low, amount of albumin probably contributed to delay the clinical diagnosis of analbuminemia or congenital hypoalbuminemia until the age of 45. The major pitfall in the diagnosis of analbuminemia is that the commonly used assays for albumin quantification in serum (based on dye-binding techniques) suffer from poor accuracy in presence of low albumin level, usually showing an overestimation of its real amount [15,16]. Serum protein electrophoresis is widely available and usually is more reliable with low serum albumin measurements, revealing the near complete absence of an albumin band (0–3 g/L) [15,16]. Immunoassays of serum albumin are likely the most accurate and often produces near-zero albumin results that are consistent with the diagnosis of analbuminemia [15,16]. However, even the most reliable immunochemical method, invariably shows the presence of non-zero concentrations of albumin [3]. For instance, in the original El Jadida patient plasma, albumin was “low” by protein electrophoresis, <10 g/L by a routine chemical technique, and <6 mg/L by an immunoassay using polyclonal antibodies and kinetic nephelometry [6]. Whether this is attributable to weak unspecific reactions or is the result of a “leaky” splicing process allowing tiny quantities of mRNA to be formed [3] still remains an open question. Therefore the final diagnosis of this rare condition requires the identification of the causative mutation within the *ALB* gene.

## 3. Experimental Section

### 3.1. Immunoblotting

Abundance of albumin was examined with the use of Western blot. Briefly, 5 μg of serum was loaded on a 10% polyacrylamide/sodium dodecyl sulfate gel and subjected to separation by electrophoresis. Proteins were electroblotted onto polyvinylidene fluoride membrane (Hybond, GE Healthcare). Antibody against human ALB was used (Bethyl, Montgomery) as well as a positive control for ALB (Baxter, Germany). Positive immunoreactivity was detected with chemiluminescence substrate (ECL detection reagent, GE Healthcare).

### 3.2. Mutational Analysis

The mutational analysis of the *ALB* gene was carried out following the principles outlined in the Declaration of Helsinki. After we obtained informed consent, we collected blood samples from the proband and extracted genomic DNA from whole blood. Fourteen genomic fragments of the *ALB* gene encompassing the fourteen coding exons and their intron-exon junctions [5] were PCR amplified using specific primer pairs as described by Watkins *et al*. [4]. Genomic DNA from two unrelated healthy volunteers was available as a control. PCR amplification and HA and SSCP analysis were performed as previously described [11,12,17]. In preparation for sequence analysis, 5 μL of PCR products were cleaned up by ExoSAP-IT. (USB Corporation; GE Healthcare EUROPE GmbH). After digestion, 2.5 μL of purified PCR products were subjected to typical sequencing reactions by adding 1 μL of BigDye Terminator (Applied Biosystems) and 5 pmol of primer in a final reaction volume of 10 μL. Cycle conditions consist in a rapid denaturation (96 °C for 10 s) and annealing/extension (60 °C for 3 min) for 25 cycles, followed by a terminal extension at 72 °C for 6 min. Excess of dye terminators were removed by ethanol precipitation twice. Samples were electrophoresed on an automated DNA sequencing instrument (Applied Biosystems 3100), using 50 cm capillary arrays and POP-6 polymer. Data were analysed using the Sequencher software version 4.7 (Genecodes Corp.).

### 3.3. Two-Dimensional Electrophoresis

Two-dimensional polyacrylamide gel electrophoresis was performed using the IPG system [18]. The first dimension, isoelectric focusing, was carried out on laboratory-made gels, cast on GelBond with a 4–10 non-linear immobilized pH gradient obtained with Acrylamido buffer solutions (Fluka) and the separation was run in the Multiphor II horizontal system (Amersham Biosciences). The gel strips were then equilibrated with SDS, placed on top of vertical 10% gels, and the second dimension was carried out using a Mini PROTEAN II cell (Bio-Rad). The gels were stained with Coomassie blue.

## 4. Conclusions

The case here reported shows that analbuminemic individuals are prone to misdiagnosis or delayed diagnosis. Clinicians should consider a diagnosis of analbuminemia even when the clinical laboratory detects ALB levels higher than 10 g/L, because routine assays, based on dye binding techniques, suffer from very poor accuracy in presence of a low ALB amount, and also immunoassays and serum protein electrophoresis may reveal non-zero amounts of the protein. Our strategy, based on the combination of SSCP, HA, and DNA sequencing allows the molecular diagnosis of this rare disorder, that is always necessary because the presence of minute ALB amounts may have many causes other than a genetic lack of the protein. In the case reported here the causative mutation escaped the SSCP and HA, probably because of the large size of the mutated fragment (394 bp). As seen in the original El Jadida case, however, this mutation could be evidenced by SSCP after digestion of the appropriate restriction enzyme [6]. The identification of the defects in the *ALB* gene causing analbuminemia will contribute to shed light on the molecular basis underlying the trait.

## Figures and Tables

**Table 1:** Clinical laboratory test results on serum of the analbuminemic subject.

Analyte	Proband	Units	Normal Reference Range
albumin (relative)	1.7	%	56.8–66.2
albumin (absolute)	0.9	g/L	35.0–52.0
α1 globulins (relative)	12.3	%	3.0–5.3
α1 globulins (absolute)	6.4	g/L	2.3–3.7
α2 globulins (relative)	29.0	%	7.2–11.4
α2 globulins (absolute)	15.0	g/L	5.2–8.6
β globulins (relative)	25.5	%	8.7–12.7
β globulins (absolute)	13.2	g/L	6.4–10.0
γ globulins (relative)	31.5	%	11.0–18.7
γ globulins (absolute)	16.3	g/L	3.8–7.5
Total protein (absolute)	51,8	g/L	60.0–81.0
Total cholesterol	8.43	mmol/L	<5.0
LDL cholesterol	5.91	mmol/L	<3.0
HDL cholesterol	1.87	mmol/L	>1.0
Triglycerides	0.84	mmol/L	<2.0
Calcium	1.94	mmol/L	2.20–2.65
Phosphate	1.72	mmol/L	0.81–1.61

The values are the average of 3 independent determinations. Albumin and the other plasma proteins were quantified by densitometric analysis of the serum protein electrophoresis pattern. All the other analytes were assayed by routine clinical laboratory procedures. % stands for percentage of the total protein content.

## Abbreviations

ALB: serum albumin (protein)
*ALB*: albumin gene
FcRn: neonatal Fc receptor
